# Editorial

**DOI:** 10.1007/s11832-013-0548-x

**Published:** 2014-01-17

**Authors:** Fritz Hefti, Shlomo Wientroub

**Affiliations:** 1 Basel, Switzerland; 2 Tel-Aviv, Israel

**Looking forward to an exciting 2014**

We would like to announce that our journal, JCOR (*Journal of Children’s Orthopaedics*) will become open access (OA) as of January 2014.

Open access is about creating a more efficient way to share scientific ideas and knowledge. Open access publication is digital, online, free of charge, and free of most copyright and licensing restrictions. It gives any user the free, immediate, full-text use of digital scientific and scholarly material. It enables rapid publication, wider dissemination and increased visibility, higher citation rates and global exposure to culturally diverse opinions and practices.

Open access is a reversed model to traditional journals. Traditional publishing relies on restricting access in order to recoup the costs of the publication process, OA treats publication as the last phase of research process. The Article Publication/Processing Charge (APC) for JCOR will be paid by EPOS on behalf of all authors regardless of society membership.

JCOR will be operated in the same way as before. Authors, readers and editors will continue to use the same online-system for manuscript submission and peer review. Any article submitted to JCOR will continue to be subjected to the journal’s thorough peer-review process and will be accepted or rejected based on the reviewers’ evaluation, article’s quality and relevance. Only articles that have been formally accepted for publication according to JCOR’s high standards will be published. Articles will instantly be freely available to read, download and share from the moment they are published.

Springer will use its best practice to ensure the widest possible visibility and dissemination of the Journal, including immediate access to journal articles via PubMed Central and PubMed without embargo.

All articles published in the Journal will be published under the Creative Commons Attribution license (CC-BY), which is industry ‘best practice’ and meets requirements by research funders such as RCUK, Wellcome Trust, US NIH and many academic and clinical institutions.

We are looking forward to your continuous support in our journal.

Happy New Year!

Looking forward to an exciting 2014.

